# Exploring Selective Exposure and Confirmation Bias as Processes Underlying Employee Work Happiness: An Intervention Study

**DOI:** 10.3389/fpsyg.2016.00878

**Published:** 2016-06-15

**Authors:** Paige Williams, Margaret L. Kern, Lea Waters

**Affiliations:** Centre for Positive Psychology, Melbourne Graduate School of Education, The University of Melbourne, MelbourneVIC, Australia

**Keywords:** work happiness, attitudes, psychological capital, organization culture, organizational virtuousness, selective exposure, confirmation bias

## Abstract

Employee psychological capital (PsyCap), perceptions of organizational virtue (OV), and work happiness have been shown to be associated within and over time. This study examines selective exposure and confirmation bias as potential processes underlying PsyCap, OV, and work happiness associations. As part of a quasi-experimental study design, school staff (*N* = 69) completed surveys at three time points. After the first assessment, some staff (*n* = 51) completed a positive psychology training intervention. Results of descriptive statistics, correlation, and regression analyses on the intervention group provide some support for selective exposure and confirmation bias as explanatory mechanisms. In focusing on the processes through which employee attitudes may influence work happiness this study advances theoretical understanding, specifically of selective exposure and confirmation bias in a field study context.

## Introduction

As the nature of business has shifted from a concentration on scarce financial capital to a concentration on scarce human capital (Bartlett and Ghoshal, 2002), people have become of strategic importance in today’s information and knowledge-driven society. In this modern economy, value is created through intangibles such as such as intelligence, creativity, and personal factors such as warmth rather than physical mass (Quah and Coyle, 2002). Further, there is a growing expectation from employees that organizations will take an active role in supporting their well-being, and this has become an important point of competitive advantage for organizations in the employment market (Martin et al., 2005). As such, organizations are recognizing the importance of intentionally supporting and fostering employee well-being if they are to access the full capacity of their human capital and perform well (Van De Voorde et al., 2012).

Positive psychology (PP) scientifically studies optimal functioning in individuals, groups, and institutions (Gable and Haidt, 2005). The application of PP in work contexts has been pursued in two complementary fields: Positive Organizational Scholarship (POS) and Positive Organizational Behavior (POB). POS is defined as “the study of especially positive outcomes, processes, and attributes of organizations” such as organizational virtues (OVs) and peak performance (Cameron et al., 2003, p. 4). POB focuses upon psychological capacities such as hope, optimism, self-efficacy and resilience (Luthans and Church, 2002; Luthans et al., 2007b). POS examines organizational phenomena, whereas POB is concerned with the cultivation of positive psychological states within individual employees. A growing body of research from these two fields suggests that employee well-being benefits both individuals and organizations. However, there has been less research exploring ways in which *individual* and *organizational* growth can be supported by organizations in ways that are mutually beneficial. This has led to a recent call for greater integration between the two fields (Youssef and Luthans, 2011).

Using the Inside-out Outside-in (IO-OI) model (Williams et al., 2016, Unpublished), the current study responds to this call by exploring two processes underlying the reciprocal and synergistic influence of individual psychological capital and organizational culture on employee well-being at work. In identifying and explaining the processes involved in these relationships, this paper also responds to calls for researchers to understand more about the mechanisms underlying PP interventions (Lyubomirsky and Layous, 2013), particularly within the work context.

### Well-being at Work

For most adults work represents half of waking life (Wrzesniewski et al., 1997). Work can provide individuals with opportunities to develop their capabilities (Ulrich and Smallwood, 2004), use their strengths (Buckingham and Clifton, 2001; Peterson and Seligman, 2004) and flourish (Keyes, 2002; Huppert, 2009). In doing so, work influences how a person feels and functions, either positively or negatively. Evidence suggests that optimal levels of well-being at work are associated with desirable outcomes for employees and organizations. For instance, well-being in employees is linked with greater engagement (Harter et al., 2003), organizational citizenship behaviors (Ilies et al., 2009a) and overall career success (Boehm and Lyubomirsky, 2008). Further, well-being at work spills over to other life domains (Sirgy et al., 2001; Ilies et al., 2009b) and has been linked with lower health risk behaviors and improved mental health (Wilson et al., 2004). For organizations, employee well-being has been related to customer satisfaction (Giardini and Frese, 2008), productivity, presenteeism, and effort at work (Keyes, 2005; Sears et al., 2013), lower voluntary turnover (Wright and Bonett, 2007), and fewer sick days (Keyes, 2005). Further, organizations that develop employee well-being receive a positive return on investment through reduced absenteeism and compensation claims (PricewaterhouseCoopers, 2014). Thus, employee well-being has benefits for both the individual and the organization; as such it is in the interest of organizations to intentionally develop it.

Workplace well-being has been defined in a number of ways (e.g., Cotton and Hart, 2003; Page and Vella-Brodrick, 2009; Schulte and Vainio, 2010; Sharma and Sharma, 2015). One key component of well-being is happiness, which Lyubomirsky (2007, p. 32) defines as “the experience of joy, contentment, or positive well-being, combined with a sense that one’s life is good, meaningful, and worthwhile.” Fisher (2010) suggests that existing measures of work happiness such as job satisfaction (Locke, 1976), positive affect (Fisher and Boyle, 1997), and thriving and vigor (Spreitzer and Sonenshein, 2004) are limited by a focus on the individual level of analysis, reference only to pleasant experiences or judgments, and a focus on core job task performance. She proposes an holistic, higher-order approach to conceptualizing work happiness comprising: (a) engagement with the work itself, (b) satisfaction with the job, and (c) feelings of affective commitment to the organization as a whole. Fisher’s model of work happiness parallels recent advances in well-being theory, which suggest that well-being is multidimensional in nature (e.g., Forgeard et al., 2011; Seligman, 2012; Huppert and So, 2013) and has received some empirical support (Williams et al., 2015). The IO-OI model adopts Fisher’s model of work happiness as the outcome of interest.

### The Inside-Out Outside-In Model of Work Happiness

**FIGURE 1:**
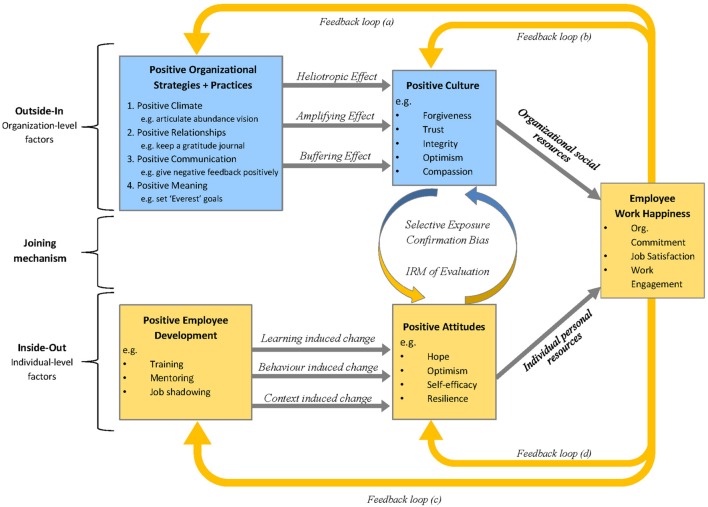
**The Inside Out-Outside In (IO-OI) model: a dual approach process model to developing happiness at work**.

The IO-OI model (Williams et al., 2016, Unpublished) is a dual approach process model that proposes that work happiness is influenced by factors ‘inside’ the employee and factors ‘outside’ of the employee (see **Figure 1**). Factors inside the employee are those that influence an employee’s experience of work and that cannot be separated from the individual, such as attitudes, values, beliefs, emotions, and behaviors. Factors outside of the employee are defined as those that influence an employee’s experience of work and that are discrete to the individual, such as the organizational culture, work climate, job characteristics, manager/supervisor, colleagues, and the physical work environment. For instance, targeting positive employee development through training, mentoring, and job shadowing provides an ‘inside-out’ approach, as it aims to directly influence attitudes, beliefs, and behaviors in order to positively impact the individual’s work experience. In contrast, a positive strategies and practices framework aimed at developing a positive organizational culture provides an ‘outside-in’ approach, as it aims to influence the environment in which the individual operates to positively impact the individual’s work experience.

Evidence suggests that both approaches enable and support the development of work happiness, but a combination of both inside-out and outside-in approaches may offer the greatest benefit (Williams et al., 2015). The IO-OI model further suggests that synergistic relationships exist between the inside-out and the outside-in approaches. This occurs via three processes: (1) the iterative reprocessing of evaluations, (2) selective exposure, and (3) confirmation bias The purpose of the current study is to examine the influence of the latter two processes – selective exposure and confirmation bias – as explanatory mechanisms for the associations between employee positive attitudes [conceptualized as psychological capital (PsyCap), Luthans et al., 2006], perception of virtues in the organization culture (conceptualized as OV, Cameron et al., 2004), and levels of work happiness (WH, Fisher, 2010).

### Selective Exposure and Confirmation Bias

*Selective exposure* is the phenomenon whereby people choose to focus on information in their environment that is congruent with and confirms their current attitudes in order to avoid or reduce cognitive dissonance (Festinger, 1962). It comprises three sub-processes: (a) selective exposure, through which people avoid communication that is opposite to their existing attitude; (b) selective perception, when people are confronted with unsympathetic material, either they do not perceive it or they make it fit for their existing opinion; and (c) selective retention, when people simply forget attitude-incongruent information (Klapper, 1960).

In addition to filtering the information that is attended to, individuals may also actively seek out and assign more weight or validity to information that supports their current attitude. This is known as *confirmation bias*, and impacts the way in which people search for, interpret, and recall information (Wason, 1960). Selective exposure and confirmation bias have been found to impact decision making in a number of settings including health (Nickerson, 1998), politics (Hart et al., 2009), and scientific research (Hergovich et al., 2010). However, they have not been explored as processes that explain how employee attitudes and perception of organization culture interact to influence levels of work happiness.

### Positive Employee Attitudes

Attitudes serve as a mental heuristic that help us navigate our environment effectively and efficiently by removing the need for us to evaluate and make decisions about each new object encountered. An attitude is defined as “an evaluation of an object of thought” (Bohner and Dickel, 2011, p. 392) and have been found to help categorize objects (Smith et al., 1996), support decision-making ease (Blascovich et al., 1993), and decision-making quality (Fazio et al., 1992). Attitudes of organization members assist them to evaluate their job role, behaviors of managers and colleagues, organization policies, and the organization environment as a whole. Attitudes influence and are manifest through an individual’s thoughts, feelings, and behaviors. As such, the development of positive attitudes in employees is likely to increase ‘inside’ personal resources such as the frequency of positive affect; positive core-self evaluations linked to resiliency, and levels of efficacy through an individual’s sense of their ability to successfully control their environment. Positive attitudes may also influence perception of ‘outside’ organizational resources through a member’s ability to evaluate positive practices in the organization culture.

In this study, we conceptualize positive attitudes as psychological capital (PsyCap, Luthans et al., 2006), defined as, “an individual’s positive psychological state of development” (Luthans et al., 2007b). PsyCap has been defined as a ‘resource bank’ that enables successful performance, response to challenges and that supports people to flourish in multiple life domains, including work, relationships and physical health (Hobfoll, 2002; Lyubomirsky et al., 2005). PsyCap comprises four elements – hope, efficacy, resilience, and optimism – that function together. PsyCap has been shown to lead to higher performance outcomes in the workplace (Luthans et al., 2010). It is suggested that four mechanisms underlie this synergistic relationship: (1) positive expectations about future outcomes enabling higher motivation, (2) the development of multiple pathways to achieve goals, (3) promoting a positive response to setbacks, and (4) reinforcement of greater extra effort from individuals (Luthans et al., 2010, p. 48).

Although PsyCap is often thought of as a resource, we propose that it can also be understood as attitudes. The four elements of PsyCap reflect the ways in which attitudes influence and are manifest (i.e., through an individual’s thoughts, feelings, and/or behaviors). For example, an organization member with a hopeful ‘evaluation of an object of thought’ or attitude toward meeting a work target, experiences high levels of goal-directed energy (feelings), can generate multiple pathways (thoughts) and as such make plans to meet their goals (behaviors; Snyder et al., 1996). An optimistic attitude is one of high motivation (feelings) driven by positive future expectations (thoughts; Scheier and Carver, 1985). Self-efficacious attitudes comprise an individual’s confidence (feelings/thoughts) in their motivation, skills, and cognitive resources to successfully meet the demands made of them (Stajkovic and Luthans, 1998), which results in them making greater effort (behaviors). An attitude of resilience is characterized by the ability to ‘bounce back’ from challenging situations (feelings, thoughts; Masten and Reed, 2002, p. 74) and in doing so, demonstrate a positive response to setbacks (thoughts/behaviors).

The four elements of PsyCap have been found to be open to development and impacted through interventions (Youssef and Luthans, 2007), suggesting that it is a valid model to use when fostering positive attitudes in employees. On this basis, we propose that PsyCap and its elements can be understood as attitudes, and use it as the conceptualization of positive attitudes in this study.

### Attitudes, Selective Exposure, and Confirmation Bias

In theory, the attitudes of employees influence the processes of selective exposure and confirmation bias as they search for and give more weight to information that is congruent with their current attitudes. As such, it is likely that employees with positive attitudes will more easily notice the positive behaviors of colleagues and the positive practices put in place by their organization because these aspects of the organization environment are congruent with their positive attitude (selective exposure). The member may then place more validity on that information which further reinforces their positive attitude (confirmation bias), making it more likely that they will notice other positive attitude-congruent aspects of their work environment, thus activating the cycle again.

**FIGURE 2:**
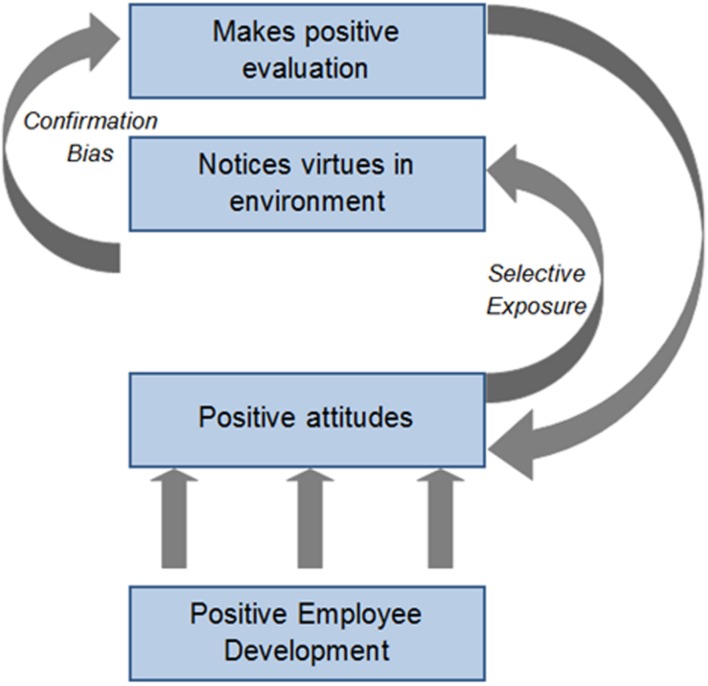
**The processes and outcomes of positive development on employee attitudes and evaluation of virtues in the organization culture**.

**Figure 2** shows the dynamic cycle that theoretically is triggered as employees develop positive attitudes and the processes of selective exposure and confirmation bias influence their perception and evaluation of the organization environment. Positive attitudes influence and are influenced by dynamic cycles that move organization members through a sequence in which positive attitudes enable them to see more virtues in the work environment (selective exposure) which reinforces their positive attitude toward the organization (confirmation bias) which in turn allows them to see more virtues (selective exposure). As such, higher levels of positive attitudes support a positive selective exposure process, in which employees focus on information in their environment that is congruent with and confirms their current positive attitudes in order to avoid or reduce cognitive dissonance. As such, they see more virtuous behavior and practice in their organizational environment.

With ongoing environmental stimuli, the cycle is likely to continue over time as selective exposure and confirmation bias influence the attention and weight individuals give to positive behaviors and practices in the organization environment. This theoretically establishes an upward spiral, whereby attitudes toward the organization become more positive as virtues are more easily perceived, seen, and acted upon. Thus the processes of selective exposure and confirmation bias support members with positive attitudes to recognize and reflect on behaviors and practices from a positive, virtues-based perspective. The positive dynamic spiral as shown in **Figure 2** may lead to higher levels of work happiness through the individual personal resources and organizational social resources that it creates.

Equally, we propose that this reinforcing selective exposure-confirmation bias spiral can work in the inverse direction, such that employees holding negative attitudes will selectively recognize and reflect on negative aspects of the organization, resulting in lower levels of work happiness. Further, as a dynamic system, employees might shift between positive and negative spirals at different times, resulting in variable levels of workplace happiness.

Moving beyond the individual, such cycles may impact the organization more broadly. Positive attitudes in a single employee may have little impact, but when spread across many employees, a tipping point may be reached through which collective change is triggered, increasing the capacity for virtuousness across the whole system. Haidt et al. (2000, Unpublished) elevation proposition suggests that when we perceive virtuous behavior in others, we are motivated to behave virtuously ourselves. Thus, as employees develop positive attitudes they become more open to the seeing virtuousness in the organization culture, because it is congruent with their existing attitude. This is then affirmed and confirmed through the processes of selective exposure and confirmation bias and as a result they perceive more virtuousness in others and evaluate other people’s behaviors from a virtues perspective. Haidt’s work suggests that by seeing more virtues in others, organization members may ‘elevate’ their actions and behave more virtuously themselves. The elevation proposition explains how the processes of selective exposure and confirmation bias may contribute to increasing the capacity for virtuousness at the collective level, thus building organizational social resources leading to increased work happiness (Williams et al., 2016, Unpublished).

### The Current Study

The current study used data from a quasi-experimental study to test the proposition that positive attitudes in employees trigger a dynamic positive loop that influences their perception and evaluation of the organization culture and overall work happiness through the processes of selective exposure and confirmation bias.

In the larger study, a group of school staff members completed questionnaires at three time points (pre-intervention, immediately post-intervention, 8 weeks post-intervention). After the first assessment, most of the staff members completed a training program that focused on skills to develop and sustain positive attitudes (PsyCap), with the remaining staff members constituting a control group. To consider selective exposure and confirmation bias as processes underlying work happiness, analyses here primarily focus on those who completed the training. However, theoretically, something is needed to begin the positive spiral. The intervention was intended to be this trigger. Thus, we also compare scores between the treatment and control group, as a preliminary test that the intervention is triggering change.

To operationalize the selective exposure process, participants were asked to reflect upon the organization culture and rate it on the five factors from the Organizational Virtuousness Scale (optimism, integrity, forgiveness, compassion, trust; Cameron et al., 2004) and their opposite (e.g., for the trust factor, the opposite is dishonesty). To operationalize the confirmation bias process, participants completed a sentence completion task immediately after the culture rating (CR) measure in the survey battery, in which the respondent completed a number of sentence ‘stems’ with their own sentence ‘tails.’ For example, the sentence stem (SS) ‘at Organization X we are expected to…’ to which the participant provided their individual sentence ending or ‘tail.’

Applying the processes of selective exposure and confirmation bias to developing work happiness, the study tests three hypotheses:

H_1_: Based on selective exposure, respondents with more positive attitudes (PsyCap) will evaluate the organization culture more positively within and over time.
H_2_: Based on confirmation bias, respondents who evaluate the organization culture more positively will provide more positive sentence tails within and over time.
H_3_: Respondents who evaluate the organization culture more positively *and* who provide more positive sentence tails will have higher levels of work happiness.

## Materials and Methods

### Participants

This study was part of a larger quasi-experimental design. In the full design, a group of 69 employees of a large independent school in Victoria, Australia participated in this study; 32 were members of teaching staff (18 females, 14 males) and 37 were employed in non-teaching roles (21 females, 16 males). The intervention group (*n* = 51; 27 females, 24 males) comprised participants who completed a 3-days PP training-based intervention that was part of an ongoing organization-wide program at the research site. For comparison, employees at the research site who had not taken part in the training intervention in previous years were identified (*n* = 59) and invited by email to participate in the study by completing a series of questionnaires. Eighteen individuals (12 females, six males; four teaching, 14 non-teaching roles) agreed to complete the measures and are considered the control group. Demographic information at times 1–3 are shown in **Table 1**.

**Table 1:** Demographic data for the samples at each time-point.

	Time 1		Time 2		Time 3	
	Treatment (*n* = 51)	Control (*n* = 18)	Treatment (*n* = 51)	Control (*n* = 11)	Treatment (*n* = 38)	Control (*n* = 9)
**Role:**						
Teaching	24	4	24	2	24	2
Non-teaching	27	14	27	9	14	7
**Gender:**						
Male	27	6	27	3	18	3
Female	24	12	24	8	20	6
**Age:**						
25–34 years	3	3	3	3	2	3
35–44 years	12	2	12	0	9	0
45–54 years	15	5	15	3	11	2
55–64 years	17	7	17	4	13	3
65+ years	4	1	4	1	3	1
**Tenure:**						
New this year	4	0	4	0	2	0
1–5 years	9	2	9	2	6	2
6–10 years	6	4	6	2	4	1
11–15 years	6	3	6	2	3	2
16–20 years	25	9	25	5	22	4
20+ years	2	0	2	0	1	0

All 51 participants in the intervention group completed the questionnaire at times 1 and 2; 38 (73%) completed it at time 3. For the control group, all 18 completed the questionnaire at time 1, 11 (64%) at time 2, and 9 (50%) at time 3. *t*-test and chi square compared the 48 individuals who completed the questionnaire at all three time points to the 22 individuals who only completed the measures at one or two occasions. There were no significant differences in terms of demographics (age, gender, role in school, hours worked, tenure status) or in terms of time 1 PsyCap, OV, or work happiness.

### Materials

The intervention group completed a training course, which comprised three consecutive days of approximately 6 h training contact time. Training materials were delivered via a mix of lecture and small group (2–4 people) activities, within groups of approximately 14–16 individuals. Each group was facilitated by two trainers, both of whom had received specialist training from the program authors to deliver the training materials. The program was based on materials developed by staff at the University of Pennsylvania’s Positive Psychology Center and draws from the relevant theories and research underpinning the four elements of PsyCap. Examples from the training include: (1) participants are taught how to dispute negative thinking patterns with more optimistic perspectives, to foster optimism and hope; (2) participants learn about the ABC Model of cognitive behavioral therapy (Ellis, 1957) and how to identify deeply held beliefs that may be driving unhelpful thought patterns and behaviors to build resilience; and (3) at the end of each topic, participants identify how they could use the skill or knowledge taught in their personal and professional lives to build efficacy.

### Procedure

The intervention group was invited to complete a questionnaire at three time points: prior to day one of the training intervention (time 1), at the end of the final day of the intervention (time 2), and 8 weeks after the intervention ended (time 3). Completion of the survey was voluntary, which was outlined in a plain language statement provided to participants at time 1 and reinforced in the verbal preamble given by the researcher prior to the first survey. Before completing the survey at time 1, participants created a unique user name, which they used to complete the surveys again at times 2 and 3. This ensured anonymity whilst enabling the marrying of results across the three measurement occasions.

For the intervention group, the survey was available in hard copy at times 1 and 2. The responses were subsequently inputted manually by the researcher. At time 3 the survey was available online via the independent survey hosting website Survey Monkey^1^ and a link to the survey was sent to the intervention group via the research site’s email system. The survey was available for a period of 10 days. Regular reminders, via email and verbal announcements at daily staff briefings were made by the researcher throughout that time to encourage participation. The control group completed the measures at the same time as the intervention group via Survey Monkey. A link to the survey was sent to them at each time point via the research site’s email system.

### Measures

The survey combined pre-established scales to measure positive attitudes (PsyCap), perception of the virtues present in the organization (OV). The work happiness scale used in Williams et al. (2015) was tested and refined for the current study, and measures for selective exposure and confirmation bias were developed by the authors.

#### Positive Attitudes

It were measured through the 24-items self-rated Psychological Capital Questionnaire (PCQ; Luthans et al., 2007a), which has been tested in samples from service, manufacturing, high-tech, military, and education sectors and across national cultural settings. In the PCQ, each of the four PsyCap factors is measured by six-items adapted from other scales. Example items include: “There are lots of ways around any problem” (hope); “When things are uncertain for me at work, I usually expect the best” (optimism); “I usually take stressful things at work in stride” (resilience); and “I feel confident presenting information to a group of colleagues” (self-efficacy). Items are scored on a six-point Likert scale from “strongly disagree” (1) to “strongly agree” (6), and are averaged together to represent the individual’s level of PsyCap (24-items, Cronbach’s α_t1_ = 0.89, α_t2_ = 0.85, α_t3_ = 0.88).

#### Perception of the Virtues Present in the Organization

The Organizational Virtuousness Scale (Cameron et al., 2004) was used to measure the perception of virtues present in the organization (OV). Work by Cameron et al. (2004) resulted in a five factor model which suggests that OV comprises: (1) organizational forgiveness, (2) organizational trust, (3) organizational integrity, (4) organizational optimism, and (5) organizational compassion. Items include: “we try to learn from our mistakes here, consequently missteps are quickly forgiven” (forgiveness); “people trust the leadership of this organization” (trust); “this organization demonstrates the highest levels of integrity” (integrity); “we are optimistic that we will succeed, even when faced with major challenges” (optimism); and “this organization is characterized by many acts of caring and concern for other people” (compassion). Each of the 15-items is scored on a six-point Likert scale from “strongly disagree” (1) to “strongly agree” (6), with higher scores indicating a greater perceived presence of that dimension of OV. The items are averaged together to represent an individual’s explicit OV score (15-items, α_t1_ = 0.96, α_t2_ = 0.93, α_t3_ = 0.95).

#### Work Happiness

It was defined using Fisher’s (2010) model, which comprises (1) engagement with the work itself; (2) satisfaction with the job, including contextual features such as pay, co-workers, supervision, and environment; and (3) feelings of affective commitment to the organization as a whole. As a single measure of this model does not currently exist, Williams et al. (2015) combined three validated scales to represent the three factors: the nine-items Utrecht Work Engagement Scale (UWES-9; Schaufeli and Bakkar, 2003) for work engagement; the eight-items Job in General Scale (JIG; Russell et al., 2004) for job satisfaction and a nine-items positively phrased version of the 15-items Organizational Commitment Scale (Mowday et al., 1979) for affective commitment. To further refine this measure, Exploratory Factor Analysis (EFA) and Confirmatory Factor Analysis (CFA) were used to test the items, which suggested 22-items best captured Fisher’s model (see Appendix A for details). Items were normalized to unity, and then averaged together to represent an individual’s work happiness at each time point (α_t1_ = 0.95, α_t2_ = 0.86, α_t3_ = 0.91).

#### Selective Exposure

There are a variety of techniques for measuring selective exposure including retrospective reports, behavioral intentions, observation of behavior and aggregate measures of behavior over time (see Clay et al., 2013 for a critical review). We were interested to examine whether a respondent’s positive/negative attitudes influenced the information they noticed, remembered and recalled about the culture of the organization. A retrospective report technique was used in which participants were asked to reflect on their organization and rate how much they noticed a specific quality was present in the culture on a scale of 0 and 100 which could be thought of as percentage out of 100 (0 = not at all, 50 = sometimes/ some areas, 100 = strongly evident). The specific qualities they were asked to rate included the five factors from the Organizational Virtuousness Scale (Cameron et al., 2004) and their opposite, (e.g., for the OV factor optimism, the opposite is pessimism). For the purpose of analysis, the ratings mean for the OV opposites (i.e., negative qualities) was deducted from the ratings mean for the OV factors (i.e., positive qualities) to give an overall positive-to-negative qualities rating for each time-point. The possible range of ratings was therefore -100 to 100, with a higher positive rating score indicating more positive qualities about the culture were noticed, remembered and recalled (α_t1_ = 0.85, α_t2_ = 0.87, α_t3_ = 0.89).

#### Confirmation Bias

It was measured through a sentence completion task that immediately followed the CR measure. Sentence completion tasks are a projective and deliberative form of attitude measure that involves the respondent completing a number of SSs (Vargas et al., 2004). Interpretation-based measures such as these examine the extent to which motives and worldviews are projected onto stimuli, thus giving an insight to individual attitudes. We considered confirmation bias to be present when the valence pattern matched (i.e., positive results in the CR measure will lead to more positive sentence tails in this measure).

Guided by extant literature (Berman and Miner, 1985; Stahl et al., 1985) 15 SSs relating to the host organization were developed (see Appendix 1). Examples include: ‘Organization X is…’; ‘At Organization X we are expected to…’ and ‘The culture at Organization X ….’ Two trained coders then rated each response (2,235 ratings each) as negative (0), neutral (1), or positive (2)^2^. Based upon guidelines by Landis and Koch (1977), Cicchetti (1994), and Bland and Altman (1999), there was substantial inter-rater agreement according to Cohen’s kappa (Cohen’s κ = 0.752), and there was excellent agreement according to the intraclass correlation (ICC) using a two-way mixed, consistency, average measure (ICC = 0.939, 95% confidence interval = 0.934, 0.944 (Hallgren, 2012). The average of the two ratings was used as the final confirmation bias score for each respondent.

### Data Analyses

First, to test the efficacy of the intervention in influencing positive attitudes (PsyCap) and perceptions of virtues in the organization (OV), standardized *t*-tests were conducted for the treatment and control group between time 1 (the beginning of day 1 of the training intervention) and time 2 (the end of the final day of the training intervention).

Hypotheses were then tested using the intervention group only. Descriptive statistics, correlation, and regression analyses were used to examine associations across the three time points among PsyCap and CRs (hypothesis 1), and CRs and SSs (hypothesis 2). To explore the interaction effect of CRs and SS (hypothesis 3), four groups were created based on tertile splits on CRs and SSs: CR+SS high (above 67% in both constructs); CR high (above 67% in CRs); SS high (above 67% in SSs); CR+SS low (below 33% in both constructs). A one-way ANOVA compared the mean scores of work happiness at each time point across these groups. In addition, an interaction variable of CR and SSs was created and regression analysis examined its effect on work happiness.

## Results

### Efficacy of the Intervention

First, to test whether the intervention sufficiently triggers the change process, the intervention group ought to show higher levels of PsyCap and OV post-intervention compared to the control group. The levels of change between the treatment and control group in their PsyCap and OV were not significant.

The control group was small in size (*n* = 16), such that this comparison was under-powered. In addition, the control group only completed the questionnaires and did not participate in an alternative intervention. To further test the intervention, we conducted a supplemental analysis with previously obtained data set. Eighty-nine participants completed the PsyCap measure (but not the OV measure) before and after the same intervention conducted in the previous year. Replicating the analysis with the current intervention group, results showed that PsyCap significantly increased from time 1 (pre-intervention) to time 2 [post-intervention; *t*(88) = 3.41, *p* = 0.001]. This provides preliminary support that the intervention triggers change in participants, though additional testing with larger samples is needed.

### Hypothesis Testing

**Table 2** shows the means, standard deviations, and correlations for the intervention group amongst PsyCap, OV, CRs, SSs and work happiness, within and across the three measurement occasions. Hypothesis 1 predicted that respondents with higher levels of PsyCap would rate the organization culture more positively within and over time and was partially supported. PsyCap at time 1 was significantly related to CRs at time 2 (*r* = 0.30, *p* = 0.04) and time 3 (*r* = 0.32, *p* = 0.05) but was not significantly related to CRs at time 1(*r* = -0.02, *p* = 0.90). PsyCap at times 2 and 3 were not related cross-sectionally or over time to ratings of the organization culture.

**Table 2:** Mean, standard deviation, and correlation for PsyCap, OV, culture ratings, sentence stems, and work happiness within and across three measurement time-points for the intervention group.

	Variable	*N*	*M*	*SD*	1	2	3	4	5	6	7	8	9	10	11	12	13	14	15
1	PsyCap T1	51	5.09	0.35	1														
2	PsyCap T2	51	5.24	0.39	0.71ˆ**	1													
3	PsyCap T3	39	5.07	0.40	0.36ˆ*	0.47ˆ**	1												
4	OV T1	50	5.38	0.54	0.36ˆ**	0.21	0.34ˆ*	1											
5	OV T2	48	5.59	0.42	0.36ˆ*	0.44ˆ**	0.43ˆ*	0.61ˆ**	1										
6	OV T3	39	5.26	0.47	0.15	0.11	0.63ˆ**	0.50ˆ**	0.43ˆ*	1									
7	Culture rating T1	48	66.61	20.37	-0.02	-0.02	0.01	0.32ˆ*	0.27	0.01	1								
8	Culture rating T2	48	76.60	17.54	0.30ˆ*	0.22	0.2	0.53ˆ**	0.55ˆ**	0.15	0.70^∗∗^	1							
9	Culture rating T3	39	66.97	19.21	0.32ˆ*	0.13	0.32	0.47ˆ**	0.28	0.39ˆ*	0.54^∗∗^	0.62^∗∗^	1						
10	Sentence stems T1	50	1.73	0.22	0.18	0.23	0.26	0.40ˆ**	0.43ˆ**	0.3	0.32^∗^	0.41^∗∗^	0.44^∗∗^	1					
11	Sentence stems T2	52	1.70	0.41	0.22	0.18	0.28	0.40ˆ**	0.49ˆ**	0.19	0.18	0.34^∗^	0.27	0.46^∗∗^	1				
12	Sentence stems T3	38	1.65	0.25	-0.08	-0.13	0.08	0.16	0.14	0.44ˆ**	0.19	0.08	0.46^∗∗^	0.50^∗∗^	0.14	1			
13	Work happiness T1	52	0.92	0.07	0.36ˆ**	0.09	0.33ˆ*	0.56ˆ**	0.37ˆ*	0.36ˆ*	0.32^∗^	0.38^∗∗^	0.44^∗∗^	0.26	0.60^∗∗^	0.16	1		
14	Work happiness T2	50	0.93	0.06	0.32ˆ*	0.26	0.41ˆ*	0.44ˆ**	0.54ˆ**	0.41ˆ*	0.15	0.43^∗∗^	0.38^∗^	0.21	0.60^∗∗^	0.09	0.80^∗∗^	1	
15	Work happiness T3	39	0.94	0.07	0.05	0.15	0.46ˆ**	0.17	0.44ˆ**	0.50ˆ**	0.23	0.27	0.39^∗^	0.35^∗^	0.69^∗∗^	0.53^∗∗^	0.52^∗∗^	0.63^∗∗^	1

*PsyCap, psychological capital. Work happiness = 22-items measure.*

*^∗^p < 0.05, ^∗∗^p < 0.01.*

Hypothesis 2, which predicted that respondents who evaluate the organization culture more positively would provide more positive sentence tails cross-sectionally and over time, was supported within time-points but not across time. CRs at each time point was significantly correlated with positive SSs within the same time (time 1: *r* = 0.32, *p* = 0.03; time 2: *r* = 0.34, *p* = 0.02; time 3: *r* = 0.46, *p* = 0.01), but this relationship was not sustained over time (e.g., time 2 CR and time 3 SSs *r* = 0.08, *p* = 0.65).

**FIGURE 3:**
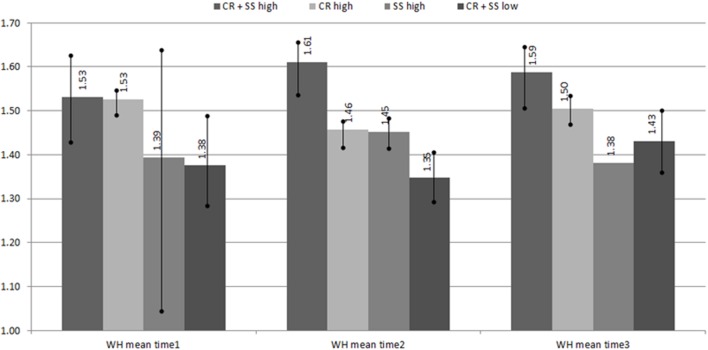
**Work happiness means and 95% confidence intervals at three time points, testing the interaction effect between selective exposure (culture ratings, CR) and confirmation bias (sentence stem completion, SS) on work happiness (WH)**.

Hypothesis 3 was tested using mean score comparison and regression analysis. **Figure 3** shows the mean scores and 95% confidence intervals for the four groups based on tertile splits of CRs and SSs at each time point. The mean comparison suggests that CRs and SSs had a small synergistic effect at times 2 and 3, but not at time. This partially supports hypothesis 3. Having high levels of both or one of the constructs influences levels of work happiness when compared to having low levels of both.

To further examine any interaction effect, we conducted a within time regression for an interaction between CR and SSs with work happiness at each time-point as the dependent variable (see **Table 3**). The *R*^2^ from the three time-points indicates that the interaction between CR and SSs explains between 15.8 and 33.4% of employee work happiness (*R*^2^ = 0.16, 0.21, 0.33, for times 1–3, respectively), with lowest levels at time 1 and highest at time 3. However, associations were not significant (time 1: *p* = 0.63, time 2: *p* = 0.26, time 3: *p* = 0.87).

**Table 3:** Regression coefficients for culture ratings, sentence stems, and an interaction variable of culture ratings and sentence stems, on work happiness across three measurement time-points.

	*r*	β	95% confidence interval	*p*
**Work Happiness Time 1**
Culture ratings	0.42	0.00	0.001, 0.006	0.008
Sentence stem	-0.02	-0.02	-0.268, 0.234	0.892
Interaction	0.08	0.00	-0.008, 0.012	0.633
**Work Happiness Time 2**
Culture ratings	0.27	0.00	0.000, 0.004	0.071
Sentence stem	0.39	0.16	0.004, 0.323	0.045
Interaction	0.21	0.01	-0.004, 0.015	0.257
**Work Happiness Time 3**
Culture ratings	0.30	0.00	0.000, 0.003	0.066
Sentence stem	0.38	0.16	0.010, 0.313	0.037
Interaction	0.03	0.00	-0.007, 0.008	0.870

## Discussion

The IO-OI model suggests that factors both inside and outside of the employee support or hinder individual well-being (Williams et al., 2016, Unpublished). To better understand when the inside and outside factors are supportive of one another versus in conflict, an understanding of mechanisms is needed. This intervention study examined selective exposure and confirmation bias as possible processes underlying associations between an inside factor [employee positive attitudes (PsyCap)], an outside factor (perception of OV), and work happiness. Results partially supported these mechanisms. PsyCap and CRs (used to conceptualize the selective exposure process) were significantly related across some time-points, but not all. CRs and positive SSs (used to conceptualize the confirmation bias process) were related cross-sectionally but not over time. Further there was some evidence of a synergistic effect between selective exposure and confirmation bias processes.

### The Influence of Selective Exposure

Selective exposure is the process through which people avoid or reduce cognitive dissonance by choosing to focus on attitude-congruent information in their environment (Festinger, 1962). Based on the selective exposure process, we expected that respondents with more positive attitudes (operationalized as higher levels of PsyCap) would choose to focus on positive aspects of the organization environment and therefore evaluate the culture more positively within and over time. This was partially supported; higher ratings of PsyCap at baseline related to more positive CRs at times 2 and 3, but not cross-sectionally. Higher levels of PsyCap at times 2 and 3 were not associated with more positive CRs within or over time. The training intervention focused on developing PsyCap skills. The results suggest that for those that already have such skills, the training triggered a positive attitude cycle, resulting in them focusing on better qualities in the organization culture over time. For those beginning at a lower baseline, it may be that the positive attitudes had not developed to a level that a positive selective exposure process was triggered. This suggests that there may be a ‘tipping point’ in the strength of attitude present in order for the selective exposure process to begin. Future research should consider strength of attitude as an independent variable of interest.

It is recognized that operationalization rarely produces perfect reflections of constructs (Duckworth and Kern, 2011). Positive attitudes were operationalized as PsyCap (i.e., hope, efficacy, resilience, and optimism), following the growing POB literature (e.g., Wright and Quick, 2009; Avey et al., 2010; Toor and Ofori, 2010; Youssef and Luthans, 2011), and selective exposure was operationalized through ratings of the organization’s culture. It may be that, although it captures four elements, the PsyCap construct is too general to trigger the selective exposure process as operationalized in this study. For example, a person may have high levels of hope and still notice un-virtuous aspects of the organization culture such as indifference, without experiencing cognitive dissonance. As a result, there would be no need to activate the selective exposure process. Further, a recent critique of the methods of measuring selective exposure suggests that, “differences in levels of specificity between initial preference measures and the information to be selected may have contributed to the somewhat conflicted findings in the literature” (Clay et al., 2013, p. 164), the result of which may be increased error variance. Future research should consider specifying initial attitudes and preferences closely in order to improve sensitivity to detecting selective exposure processes.

### The Influence of Confirmation Bias

Confirmation bias influences people to seek out and assign more weight or validity to information that supports their current attitude (Wason, 1960). Based on this process, we expected that there would be a positive association between CRs and SS valence, such that respondents who evaluated the organization culture more positively would also provide more positive sentence tails within and over time. This association was supported within time but not across time, which suggests that there is a temporal limit to the influence of confirmation bias. Much of the confirmation bias literature to date focuses on in-the-moment cross sectional timing in laboratory settings (for a review see Oswald and Grosjean, 2004). The influence of time as a factor in the process and the impact of real life settings have not been explored. Processes can occur at a micro level (within seconds and minutes) and at a macro level (over hours, days, or months). The cross sectional associations in the results of the current study point to confirmation bias being a micro rather than a macro process when occurring in a field study context. We propose that this may be connected to the dynamic nature of attitudes. The formation of attitudes is considered a critical adaptive capacity; however, of equal importance is that attitudes can be changed in light of new information and experiences (Bodenhausen and Gawronski, 2013). The purpose of the training intervention was to develop positive attitudes to trigger the selective exposure-confirmation bias processes. A defining feature of the confirmation bias process is that it is based on *current* attitudes; the cross sectional results support that this process is present. The results also suggest that the influence of the confirmation bias process does not extend to over longer time frames. As such organizations need to consider ways in which the confirmation bias process can be ‘re-triggered’ through the fostering of positive attitudes on a regular and on-going basis, in order for it to have a sustained impact on employee well-being. Further studies should investigate the temporal unfolding of confirmation bias, in both laboratory and field studies (Cialdini, 2009). Using Experience Sampling Methods might be beneficial in this regard.

### The Combined Impact of Selective Exposure and Confirmation Bias

We expected that those respondents who evaluate the organization culture more positively *and* who provide more positive sentence tails would have higher levels of work happiness. This was partially supported. Mean comparisons for four groups based on tertile splits of CRs and SSs at each time point showed some evidence of a synergistic effect at times 2 and 3. The processes of selective exposure and confirmation bias each influence the attention and weight individuals give to positive behaviors and practices in the organization environment. Together, they may create a dynamic positive cycle that influences employee perception and evaluation of the organization environment, which has been shown to influence their work happiness (Williams et al., 2015).

Regression analyses did not find a relationship between work happiness and the interaction between CRs and SSs. The sample size was small, limiting the power to find such an effect, which most likely is small in magnitude, as many factors influence a person’s happiness (Diener and Ryan, 2009). In addition, the timing of measurement time-point 1 may have influenced results, as the participants had just returned from a 7 weeks break from work (during the school summer holidays), and therefore had not been in a work environment. As such, there may not have been strong ‘current attitudes’ with which the selective exposure and confirmation bias processes could work. Future work should further examine synergistic relationships with large samples, and consider how the timing of assessments and interventions may impact responses.

### Limitations and Strengths

The results of this study need to be considered within a number of limitations. First, validated measures of selective exposure and confirmation bias in the organizational environment do not exist and so the measures used in the study were developed by the authors. As such, full validation of the measures is needed. Further, the placement of these measures next to each other in the survey battery may have led to priming effect from the selective exposure to the confirmation bias measure. As such, common method bias cannot be ruled out. The outcome variable was based on Fisher’s (2010) model of work happiness which does not currently have a single validated measure for the three inter-related domains proposed by the model. Therefore, the current study refined a measure from previous research (Williams et al., 2015), which combined three psychometrically validated measures of commitment, engagement and satisfaction, but further testing of Fisher’s model is needed. Third, the sample size was small, which limited the analyses possible. Finally, the study was conducted with employees who work in a school, which may not be generalizable to workers from other sectors.

The study also has a number of strengths. The study extends theoretical knowledge by refining a measure of Fisher’s (2010) model of work happiness. It is the first study to apply the theory of selective exposure and confirmation bias to explain associations between positive employee attitudes (PsyCap), perception of virtues in the organization culture (OV) and levels of work happiness. It is also the first study to test the theory in a field-based setting. In doing so it answers the call for researchers to understand more about the underlying mechanisms of PP interventions (Lyubomirsky and Layous, 2013).

## Conclusion

Organizations are recognizing the importance of human capital in gaining long-term competitive advantage and the role that employee work happiness plays in supporting positive employee outcomes. As such, ways in which to develop employee well-being has become an important area of focus in positive organizational research. Understanding the processes that underlie changes in work happiness in real-life settings supports the development of effective interventions to build and support employee well-being. We hope that this line of research continues to be given attention in future organizational research practice.

## Author Contributions

PW, MK, and LW: made substantial contributions to the conception or design of the work or the acquisition, analysis, or interpretation of data for the work; were involved with drafting the work or revising it critically for important intellectual content; have given final approval of the version to be published; agree to be accountable for all aspects of the work in ensuring that questions related to the accuracy or integrity of any part of the work are appropriately investigated and resolved.

## Conflict of Interest Statement

PW was employed at the research site at the time of the study.

## Footnotes

1 www.surveymonkey.com.au

2 An additional untrained coder rated all messages, but marginal distributions demonstrated that responses were biased and systematically different than the other two raters. We chose to use the two consistent trained coders.
