# Corrigendum: Exploring Selective Exposure and Confirmation Bias as Processes Underlying Employee Work Happiness: An Intervention Study

**DOI:** 10.3389/fpsyg.2017.00182

**Published:** 2017-02-13

**Authors:** Paige Williams, Margaret L. Kern, Lea Waters

**Affiliations:** Centre for Positive Psychology, Melbourne Graduate School of Education, University of MelbourneMelbourne, VIC, Australia

**Keywords:** work happiness, attitudes, psychological capital, organization culture, organizational virtuousness, selective exposure, confirmation bias

There was a mistake in the Intervention group values at Times 1 and 2 in Table 1. The correct version of Table 1 appears below. The authors apologize for the mistake. This error does not change the scientific conclusions of the article in any way.

**Table 1:** **Demographic data for the samples at each time-point**.

	**Time 1**		**Time 2**		**Time 3**	
	**Treatment (*n* = 51)**	**Control (*n* = 18)**	**Treatment (*n* = 51)**	**Control (*n* = 11)**	**Treatment (*n* = 38)**	**Control (*n* = 9)**
**ROLE**						
Teaching	24	4	24	2	24	2
Non-teaching	27	14	27	9	14	7
**GENDER**						
Male	27	6	27	3	18	3
Female	24	12	24	8	20	6
**AGE**						
25–34 years	3	3	3	3	2	3
35–44 years	12	2	12	0	9	0
45–54 years	15	5	15	3	11	2
55–64 years	17	7	17	4	13	3
65+ years	4	1	4	1	3	1
**TENURE**						
New this year	25	0	25	0	19	0
1–5 years	19	4	19	4	13	3
6–10 years	5	4	5	3	3	3
11–15 years	0	2	0	1	0	1
16–20 years	1	1	1	1	1	1
20+ years	2	7	2	2	2	1

## Conflict of interest statement

PW was employed at the research site at the time of the study. The other authors declare that the research was conducted in the absence of any commercial or financial relationships that could be construed as a potential conflict of interest.

