# Supplementary material for: Exploring Selective Exposure and Confirmation Bias as Processes Underlying Employee Work Happiness: An Intervention Study
Source: Front Psychol. 2016 Jun 15;7:878. doi: 10.3389/fpsyg.2016.00878 (PMC4908138; doi:10.3389/fpsyg.2016.00878)
Supplement: Supplementary file 1 [file Data_Sheet_1.PDF]

Appendix A

Psychometrics of the Work Happiness Measure

Fisher's (2010) model of workplace happiness includes three components: engagement with work, job satisfaction, and commitment to the organization. As no single measure of workplace happiness according to this model was available, we used three existing measures to capture the three domains: the 9-item Utrecht the 9-item Utrecht Work Engagement Scale (UWES-9; Schaufeli & Bakkar, 2003), the 8-item Job in General Scale (JIG; Russell et al., 2004), and the 15-item Organizational Commitment Scale (Mowday, Steers, & Porter, 1979). Here we provide a preliminary psychometric test of the Fisher's model.

A prior study (Williams et al., 2015) included the same measures. We combined data from that study (n = 261) and the current study (n = 69), creating a psychometric dataset that we call the *combined* sample (N=330). Using SPSS software (version 23), we randomly split the combined sample into a *training* set and a *test* set using SPSS 23, specifying an approximate 50% split. Due to missing data, 172 participants were included in the training set and 158 were included in the test set.

Using the *training* set, we first conducted an exploratory factor analysis (EFA). The Kaiser criterion (eigenvalues > 1) suggested 5 factors, whereas examination of the scree plot suggested 3 to 4 factors. We extracted 3, 4, and 5 factors. The three factor structure provided the clearest structure, except for two of the job satisfaction items that cross loaded on multiple factors. In addition, we examined inter-item reliability, testing the change in reliability if any items were removed. Based on the combination of the EFA and reliability analyses, we removed four items: "enjoyable" and "excellent" for satisfaction, "I get carried away when I am working" for engagement, and "I would accept almost any type of job

assignment in order to keep working for this organization" for commitment. This reduced the number of items in the battery to 22.

With the *training*, *test*, and full combined, we then tested confirmatory models (CFA) using the lavaan package (version .5.16, Rosseel, 2012) in R (version 3.0.3), with the items loading on their respective factors, and the factors loading on a higher order work happiness latent factor. Model fit was primarily examined using RMSEA (Root Mean Square Error of Approximation) and SRMR (Standardized Root Mean Residual Model). Values below .10 are considered acceptable; although an RMSEA below .06 combined with a SRMR below .09 are recommended (Hu & Bentler, 1999). We also report the Tucker Lewis Index (TLI) and the Comparative Fit Index (CFI) for completeness.

Table A1 summarizes the items with standardized latent factor loadings and fit indices for the training, test, and combined sets. Across the three samples, reliability for the final work happiness variable is high (α_{train} = .93, α_{test} =.92, $\alpha_{combined}$ = .92). Despite modifications, the model marginally fit the data. This provides some initial support for Fisher's model, but also suggests that either measure refinements are needed to adequately measure the higher order construct, or that the overall theory may not be correct. Fully developing a work happiness measure is well beyond the scope of the current study. We proceeded with the 22-item scale as our measure of work happiness. However the measurement error inherent to the measure should be kept in mind when interpreting the results.

Table A1

Latent factor loadings and fit indices in confirmatory factor analysis for the final 22-item scale, by sample.

Factor/items	Latent Factor Loadings		
	**Train**	**Test**	**Combo**
Satisfaction	α = .84	α = .69	α = .69
Think of your job in general: what is it like most of the time?			
Good	.61	.65	.61
Undesirable	-.84	-.66	-.77
Better than most	.56	.58	.57
Disagreeable	-.80	-.61	-.73
Makes me content	.66	.69	.67
Poor	-.80	-.79	-.79
Engagement	α = .94	α = .94	α = .94
At my work I feel bursting with energy	.81	.81	.81
At my job I feel strong and vigorous	.86	.85	.86
I am enthusiastic about my job	.89	.91	.90
My job inspires me	.84	.87	.85
When I get up in the morning I feel like going to work	.82	.81	.81
I feel happy when I am working intensely	.81	.73	.77
I am proud of the work that I do	.77	.70	.73
I am immersed in my work	.76	.74	.75
Commitment	α = .94	α = .92	α = .92
I am willing to put in a great deal of effort beyond that normally expected in order to help this organization be successful.	.71	.57	.65
I talk up this organization to my friends as a great organization to work for.	.85	.76	.82
I find that my values and the organization's values are very similar.	.79	.80	.80
I am proud to tell others that I am part of this organization	.89	.90	.89
This organization really inspires the very best in me in the way of job performance	.86	.90	.88
I am extremely glad that I chose this organization to work for over others I was considering at the time I joined.	.80	.84	.81
I really care about the fate of this organization.	.88	.67	.79
For me this is the best of all possible organizations for which to work.	.81	.75	.78
Work Happiness	α = .93	α = .92	α = .92
Satisfaction	.77	.84	.79
Engagement	.63	.78	.69
Commitment	.74	.83	.78
Fit Indices			
N	172	158	330
RMSEA<br>(90% confidence interval)	.11<br>(.10, .12)	.11<br>(.10, .12)	.09<br>(.08, .10)
Standardized Root Mean Square Residual (SRMR)	.06	.07	.07
Comparative Fit Index (CFI)	.86	.85	.90

Tucker Lewis Index (TLI)	.84	.83	.88

Note. Confirmatory factor analyses were estimated using the lavaan package (version 0.5.16) in R (version 3.0.3), using Time 1 measurement occasions. RMSEA = Root Mean Square Error of Approximation, SRMR = Standardized Root Mean Residual, CFI = Comparative Fit Index, TLI = Tucker Lewis Index. For sample, Train = training sample, Test = testing sample, Combo = combined across these 2 samples.
